# Mars Regolith Simulant Ameliorated by Compost as in situ Cultivation Substrate Improves Lettuce Growth and Nutritional Aspects

**DOI:** 10.3390/plants9050628

**Published:** 2020-05-14

**Authors:** Luigi G. Duri, Christophe El-Nakhel, Antonio G. Caporale, Michele Ciriello, Giulia Graziani, Antonio Pannico, Mario Palladino, Alberto Ritieni, Stefania De Pascale, Simona Vingiani, Paola Adamo, Youssef Rouphael

**Affiliations:** 1 Department of Agricultural Sciences, University of Naples Federico II, 80055 Portici, Italy; lgduri@libero.it (L.G.D.); nakhel_christophe@hotmail.com (C.E.-N.); ciriello.michele94@gmail.com (M.C.); antonio.pannico@unina.it (A.P.); mario.palladino@unina.it (M.P.); depascal@unina.it (S.D.P.); vingiani@unina.it (S.V.); adamo@unina.it (P.A.); 2 Department of Pharmacy, University of Naples Federico II, 80131 Naples, Italy; giulia.graziani@unina.it (G.G.); alberto.ritieni@unina.it (A.R.); 3 Staff of Unesco Chair for Health Education and Sustainable Development, 80131 Napoli, Italy; 4 Interdepartmental Research Centre on the ‘Earth Critical Zone’ for Supporting the Landscape and Agroenvironment Management (CRISP), University of Naples Federico II, 80055 Portici, Italy

**Keywords:** *Lactuca sativa* L., Mojave Mars simulant (MMS-1), compost amendment, phytotron open gas exchange growth chamber, ISRU, mineral content, photosynthetic activity, phenolic profile, space mission

## Abstract

Heavy payloads in future shuttle journeys to Mars present limiting factors, making self-sustenance essential for future colonies. Therefore, in situ resources utilization (ISRU) is the path to successful and feasible space voyages. This research frames the concept of planting leafy vegetables on Mars regolith simulant, ameliorating this substrate’s fertility by the addition of organic residues produced in situ. For this purpose, two butterhead lettuce (*Lactuca sativa* L. var. *capitata*) cultivars (green and red Salanova^®^) were chosen to be cultivated in four different mixtures of MMS-1 Mojave Mars simulant:compost (0:100, 30:70, 70:30 and 100:0; v:v) in a phytotron open gas exchange growth chamber. The impact of compost rate on both crop performance and the nutritive value of green- and red-pigmented cultivars was assessed. The 30:70 mixture proved to be optimal in terms of crop performance, photosynthetic activity, intrinsic water use efficiency and quality traits of lettuce. In particular, red Salanova^®^ showed the best performance in terms of these quality traits, registering 32% more phenolic content in comparison to 100% simulant. Nonetheless, the 70:30 mixture represents a more realistic scenario when taking into consideration the sustainable use of compost as a limited resource in space farming, while still accepting a slight significant decline in yield and quality in comparison to the 30:70 mixture.

## 1. Introduction

NASA has fixed the year 2030 as target date for a manned mission to Mars [1,2,3,4,5]. With this announcement, the agency confers a real opportunity to colonize the red planet, a scenario where space farming encompasses the success of such a long-lasting space mission. A journey to Mars requires tool inputs and food supplies, but loading the latter onto the shuttle involves serious technical limitations [1,2], without even considering that this periodical delivery of inputs is also economically and operatively unfeasible [3,4]. Self-sustenance is key for the success of future colonies; therefore, a better comprehension of in situ resources utilization (ISRU) is crucial [1,5].

Plants can sustain crew survival away from the Earth, by producing fresh food as part of their edible biomass and simultaneously contributing to several ecological services like air purification and water recycling [2,6,7,8,9], as well as sustaining the psychological wellbeing of space explorers [10,11,12]. More importantly, the selection of candidate crops for food production is done using specific criteria [13,14] such as nutritional value, plant size, adaptability to extreme environmental conditions (i.e., different conditions of gravity and temperatures), low input requirements (in terms of nutritional elements, water and light), short plant life cycle and high harvest index [9,13,15,16]. Among various potential candidate species (cereals, vegetables and tubers), lettuce (*Lactuca sativa* L.) is well ranked. Indeed, lettuce leaves are rich in antioxidant compounds and in macro- and micronutrients, which can support the human diet as part of our daily intake [17,18]. Nevertheless, the nutritional value of lettuce depends on the cultivar and its interaction with the environment [19,20,21]. Moreover, plants can be a source of health, promoting secondary metabolites such as phenols [22,23], whose formation and concentration is species and stressor dependent [22,24]. For instance, nutritional chemical eustress like moderate salinity and nutrient deficiency can positively trigger physiological responses, improving vegetables’ nutritional value [25,26,27]. 

The Mars surface is composed primarily of mafic rocks, usually basalts [28,29,30,31]. Basaltic rocks and sediments are composed of varying amounts of olivine, pyroxene, plagioclase, and vitric and lithic fragments. On Mars, these minerals are accompanied by variable amounts of iron oxides and sulfates [32], suggesting that basaltic sediments may weather physically and chemically, providing additional insights into the formation of Mars soils and dust. As for the presence of Mars organic matter, very low amounts were detected by the current survey from landers and rovers [33].

To the best of our knowledge, very few works have dealt with cultivation on Mars simulants. Among them, we must mention Gilrain et al. [34], Mortley et al. [35] and Wamelink et al. [36], with only Gilrain et al. [34] adopting diverse ratios of simulant and compost. Moreover, there are no data concerning the responsive interaction between plant qualitative traits and a Mars simulant substrate. Therefore, with the perspective of this framework, the potentialities and limitations of lettuce cultivation on the red planet have to be evaluated. For these reasons, two lettuce cultivars with different pigmentations were selected for a growth chamber experiment, using the Mojave Mars simulant (MMS-1) as a hypothetic in situ substrate resource amended with a vegetal compost, to simulate the organic waste produced during journeys on Mars. As demonstrated in a recent complementary study [37], the amendment with green compost enhanced the physicochemical and hydraulic properties of the alkaline and nutrient-poor Mars simulant, concomitantly resolving the disposal issue of organic effluents in future manned missions to Mars. Overall, the data produced in this study represent the first knowledge on the response of plants to a very extreme environment, such as that of the Mars simulant, in relation to the nutritional profile (mineral composition, antioxidant compounds and phenolic acids). This information represents a major utility for planning future space missions intent on Mars colonization.

## 2. Results

### 2.1. Yield and Physiological Parameters

**Figure 1:**
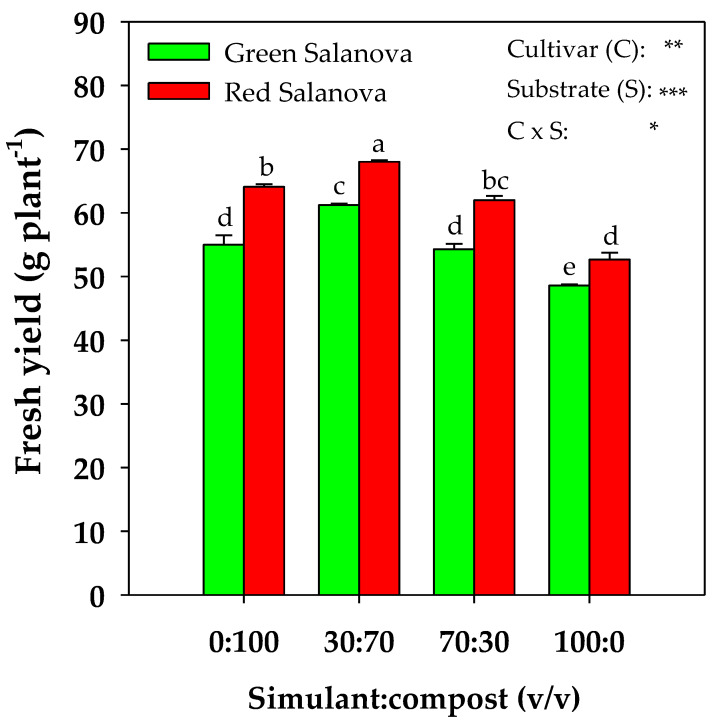
Fresh yield of green and red butterhead Salanova lettuce, as influenced by substrate mixtures (four different rates of Mojave Mars simulant:compost v:v). Different letters above bars indicate significant mean differences according to Duncan’s multiple range tests (*P* ≤ 0.05). Vertical bars indicate ± SE (standard error) of means. *, **, *** Significant at *P* ≤ 0.05, 0.01 and 0.001, respectively.

As illustrated in Figure 1, fresh yield exhibited a significant interaction (*P* ≤ 0.05) between the cultivar (C) and Mars simulant rate in the substrate (S). Both butterhead lettuce cultivars had the highest fresh yield in the 30:70 simulant:compost mixture, registering 61.2 and 68.0 g plant^−1^ fresh weight (fw) for green and red Salanova, respectively, whereas the lowest fresh yield was recorded for both cultivars in 100% simulant, ~21% lower than in the 30:70 mixture. The other two substrate mixtures (0:100 and 70:30) showed intermediate fresh yield with a different percentage reduction between the two cultivars in comparison to the highest fresh weight.

**Figure 2:**
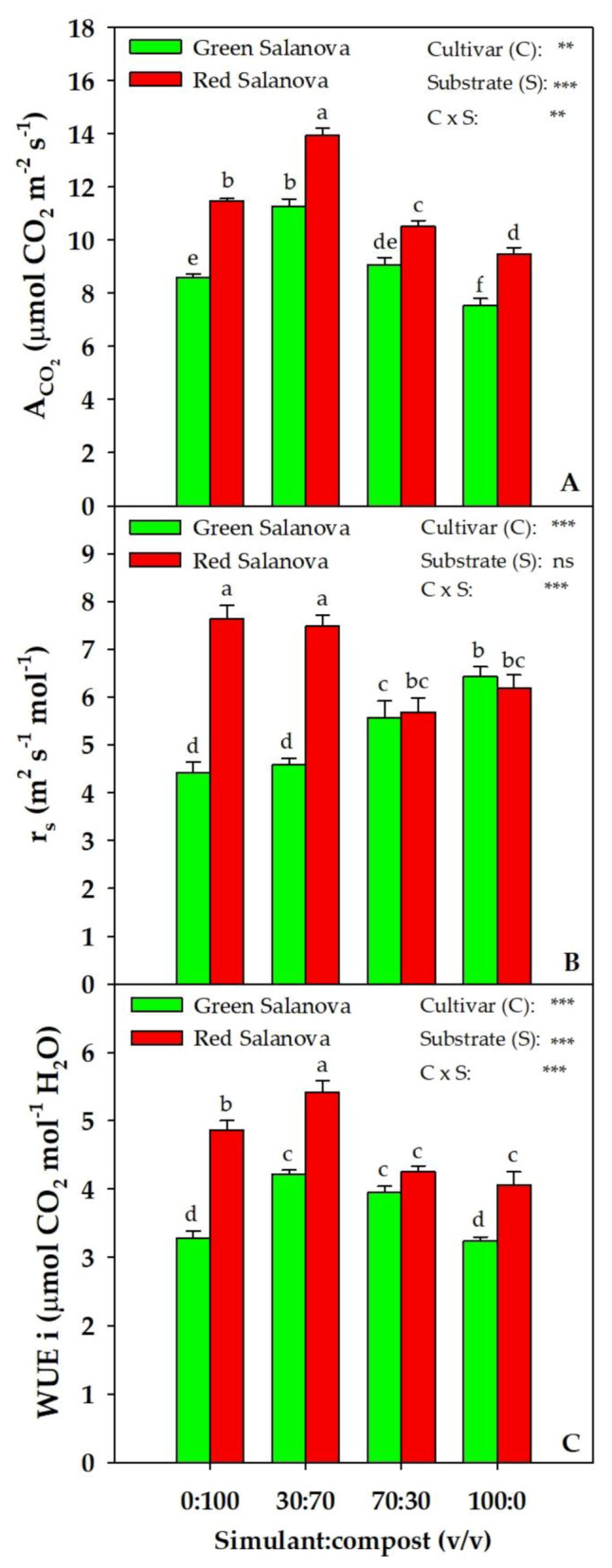
Physiological parameters: net CO_2_ assimilation rate (A_CO2_) (**A**), stomatal resistance (r_s_) (**B**) and intrinsic Water Use Efficiency (WUEi) (**C**) of green and red Salanova lettuce, as influenced by substrate mixtures (four different rates of MMS-1 simulant:compost v:v). Different letters above bars indicate significant mean differences according to Duncan’s multiple range tests (*P* ≤ 0.05). Vertical bars indicate ± SE of means. ns,**, *** Non-significant or significant at *P* ≤ 0.01 and 0.001, respectively.

All physiological measurements presented in Figure 2 showed a significant interaction (C × S). As mean effect of the simulant:compost mixture, the transpiration rate (E) was the highest in the 30:70 mixture (2.6 mol H_2_O m^−2^ s^−1^) and the lowest in both 70:30 and 100:0, with 0:100 being insignificantly different in between the three mixtures (data not shown). Noteworthily, the cultivar factor had no effect on this physiological parameter. As for the net CO_2_ assimilation rate (A_CO2_), green and red Salanova showed the highest values in the 30:70 mixture (11.3 and 14.0 μmol CO_2_ m^−2^ s^−1^, respectively) and the lowest values in 100:0 (33 and 32% lower, respectively). Stomatal resistance (r_s_) was the highest in 100:0 (6.43 m^2^ s mol^−1^) for green Salanova and in 0:100 and 30:70 (7.63 and 7.48 m^2^ s mol^−1^, respectively) for red Salanova (Figure 2). As for the intrinsic Water Use Efficiency (WUEi), the highest values were noted in 30:70 and 70:30 for green Salanova and in 30:70 for red Salanova, while the lowest values were noted in 0:100 and 100:0 for green Salanova and in 70:30 and 100:0 for red Salanova (Figure 2).

### 2.2. Shoots and Roots Mineral Composition

The analysis of shoot and root mineral contents on a dry weight basis (Table 1) showed essentially no significant differences between cultivars and no interaction between the two factors C × S. The only exception was the root nitrate concentration, which was significantly higher in green Salanova (42.9 g kg^−1^ dw), and the shoot SO_4_ concentration, which was significantly higher in red Salanova (2.5 g kg^−1^ dw). Moreover, the interaction between C × S was significant (*P* ≤ 0.05) only for the root Mg concentration, reaching the highest value of 4.3 g kg^−1^ dw in 100:0 (100% simulant) for green Salanova, whereas, for the red cultivar, the values of all mixtures, except for 0:100 (100% compost), had insignificantly different values, with an approximate mean of 3.2 g kg^−1^ dw. In contrast, there were significant differences between substrates. In 100% simulant, shoot and root mineral composition was characterized by the lowest values of nitrate (only shoot), PO_4_, K and SO_4_, and by the highest accumulation of Mg and Na. In the same substrate, Salanova shoots exhibited the highest concentration of Ca, which increased gradually with the rise in the simulant rate in the substrate (Table 1). In 100% compost, shoot and root mineral composition were characterized by the highest concentrations of Cl and K. The latter concentration reduced gradually with the increase in the simulant rate in the substrate, to register a value of 15.5 g kg^−1^ dw in the roots and 45.2 g kg^−1^ dw in the shoots (3.8- and 1.7-fold less than the other three mixtures, respectively), simultaneously accompanied by an increase in Na content in the roots (1.7 g kg^−1^ dw) and shoots (12.8 g kg^−1^ dw; four- and twofold, respectively; see Table 1). 

As for total nitrogen and nitrate expressed on a fresh weight basis (Table 2), no significant difference was found, neither for the cultivar and substrate factors mean effect nor for their interaction.

### 2.3. Total Ascorbic Acid, Total Chlorophyll and Carotenoids Content

**Figure 3:**
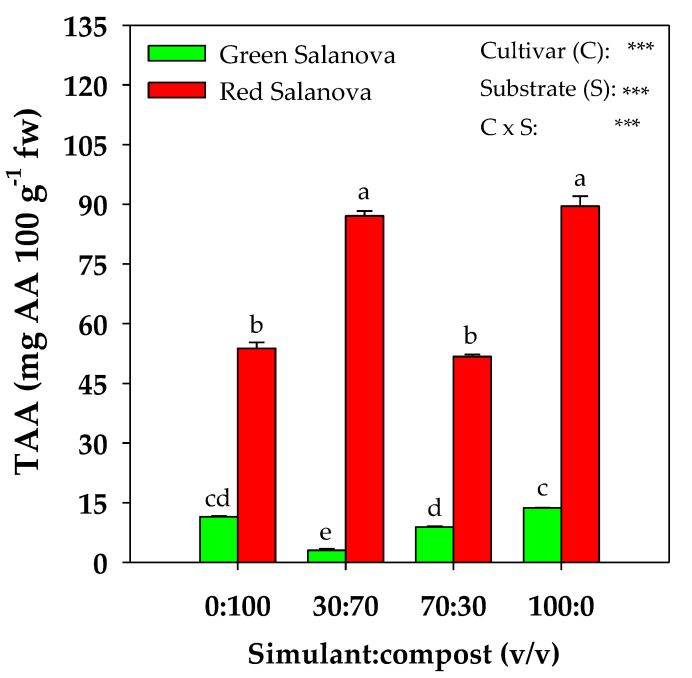
Total ascorbic acid (TAA) content of green and red Salanova lettuce, as influenced by substrate mixture (four different rates of MMS-1 simulant:compost v:v). Different letters above bars indicate significant mean differences according to Duncan’s multiple range tests (*P* ≤ 0.05). Vertical bars indicate ± SE of means. *** Significant at *P* ≤ 0.001.

As reported in Table 2, lutein and β-carotene did not exhibit any interaction between the two factors C × S, with both being significantly more concentrated in the red cultivar, and β-carotene being only influenced by the mean effect of the cultivar. In terms of the mean effect of the mixture, lutein was, significantly, the highest in the 70:30 mixture and the lowest in 100% simulant (31.7% less) (Table 2). Moreover, total chlorophyll showed the same trend as β-carotene, only being influenced by the mean effect of the cultivar, with the red cultivar registering a significantly higher content. Total ascorbic acid manifested a significant interaction C × S (Figure 3). Indeed, in the 30:70 mixture, green and red cultivars behaved differently, where green Salanova registered the lowest value of 3.0 mg AA 100 g^−1^ fw and red Salanova showed the highest value of around 87.1 mg AA 100 g^−1^ fw along with 100% regolith (Figure 3).

### 2.4. Polyphenols Content Profile

**Figure 4:**
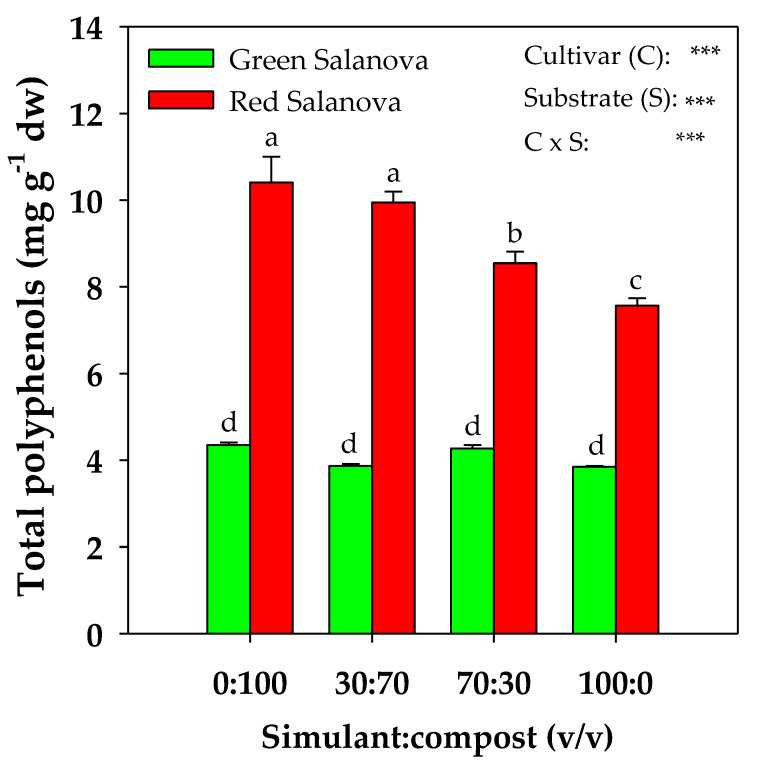
Total polyphenol content of green and red Salanova lettuce, as influenced by substrate mixture (four different rates of MMS-1 simulant:compost v:v). Different letters above bars indicate significant mean differences according to Duncan’s multiple range tests (*P* ≤ 0.05). Vertical bars indicate ± SE of means. *** Significant at *P* ≤ 0.001.

The polyphenol profiles studied in green and red Salanova are presented in Table 3. Among all the detected polyphenols, only quercetin–malonyl–glucoside showed no significant interaction between the two factors C × S. Indeed, the cultivar and substrate mean effect determined the differences, with red Salanova showing a value of 1276 μg g^−1^ dw, around 52% higher than that of green Salanova. Furthermore, in terms of the mean effect of the mixture, this phenolic compound was the most concentrated in 100% compost (1335 μg g^−1^ dw), around 63.8% higher than the average registered in the other three mixtures (Table 3). The most abundant polyphenols in both cultivars were feruloyl tartaric acid, rutin, quercetin–malonyl–glucoside, caffeoyl feruloyl quinic acid, coumaroyl quinic acid and chlorogenic acid, but in different concentrations. Chlorogenic acid content was not influenced by the substrate mixture in green Salanova (≈ 330 μg g^−1^ dw), while it was the highest in 0:100 and 30:70 mixtures for red Salanova (≈ 4780.5 μg g^−1^ dw) and decreased by 37% in 100% simulant. An opposite trend was noted for feruloyl tartaric acid, whose content in red Salanova was not influenced by the mixture (≈ 978 μg g^−1^ dw), while, in the green cultivar, the highest content was registered in 100% compost (1099 μg g^−1^ dw). As for coumaroyl quinic acid, the highest content was registered in 100% simulant for green Salanova (562.4 μg g^−1^ dw) and in the 30:70 mixture for its red counterpart (890.2 μg g^−1^ dw). Caffeoyl feruloyl quinic acid and rutin registered the highest content in 100% compost for the green cultivar (577 and 884 μg g^−1^ dw, respectively) and for the red cultivar (692 and 577 μg g^−1^ dw, respectively; Table 3). Finally, this significant interaction between C× S was also obvious for the total polyphenol content. As a matter of fact, green Salanova total polyphenol content did not vary statistically among the different mixtures, while red Salanova total polyphenol content decreased gradually with the simulant rate increase (Figure 4).

## 3. Discussion

Mars colonization can solely be realized via the adaptation of a bioregenerative life support systems (BLSSs) without umbilical support from Earth [38], by using in situ resources as much as possible and avoiding any additional reload due to technical and economic constraints [4,39]. In the present study, the utilization of MMS-1 as a plant growth substrate mixed with variable rates of compost was studied to grow two cultivars of lettuce, with the purpose being to identify suitable and sustainable simulant:compost rates, enabling future colonists to obtain a compromise between the yield and nutritional status of the produced vegetables. Caporale and co-workers [37] characterized the pure and mixed substrates from a physical, chemical, mineralogical and hydraulic point of view. They found that MMS-1 is a coarse-textured alkaline mineral substrate mainly composed of plagioclase and amorphous material with accessory minerals, including zeolite, hematite and smectite clays. Although MMS-1 can be a source of nutrients (i.e., Ca, Fe, Mg, K), it lacks organic matter, N, P and S, which can be only supplied through a compost amendment, which, in turn, enhances the main physical, chemical and hydraulic properties of the plant growth substrate.

Simulant:compost mixtures had a clear effect on Salanova lettuce yield, with the 30:70 mixture revealing the highest registered yield for both cultivars, and 100% simulant revealing the lowest yield. Similarly, a superior yield with the addition of compost to JSC Mars-1 simulant was noticed for Swiss chard [34]. In our case, such a yield response can be interpreted by the highest A_CO2_ and WUEi for both cultivars and a low r_s_ for green Salanova observed in the 30:70 mixture, alongside the lowest A_CO2_ and WUEi for both cultivars and a higher r_s_ for the green cultivar in 100% simulant. The application of organic matter had been shown to increase the concentration of chlorophylls a and b [40], and to promote net photosynthesis and water use efficiency [41]. Indeed, in this study, the best performance was observed in lettuce grown in the three mixtures containing compost that enhanced water and nutrient availability, especially in the mixture with 70% compost (30:70). Our results confirm Rouphael et al.’s [38] observations about the better yield performance and higher A_CO2_ and WUEi of red Salanova in comparison to green Salanova. This observation, in the extreme environment of extraterrestrial farming, could prove very useful, because optimized water use efficiency in an environment with low water availability, and higher CO_2_ assimilation in an abundant CO_2_ atmosphere (95%) [32,42,43,44] could be highly appreciated, especially in a BLSSs. Moreover, it was demonstrated that reduced gravity indirectly affects the surrounding environment of the plant, influencing the physiological transport of water and solutes, and gas exchange [45]. For instance, on Mars, the low gravity (1/3 of Earth’s gravity) could interact with the buoyancy-driven thermal convection, causing an increase in the boundary layer thickness with consequent biophysical limitations on the processes of gas exchange and transpiration in higher plants [45].

Simulant:compost mixtures, particularly 100% simulant and 100% compost, enhanced the accumulation of certain elements in both lettuce cultivars. Only the 30:70 mixture produced a proper accumulation of NO_3_, PO_4_ and K in Salanova shoots, associated with a good repartition between shoots and roots, which explains the higher yield of green and red Salanova obtained in this mixture. All three mixtures rich in compost showed higher shoot and root accumulation of SO_4_ in comparison to 100% simulant, which can be explained by the increasing bioavailability of the anion with the increasing rate of compost in the growth substrate [37]. Furthermore, red Salanova significantly accumulated more SO_4_ than its green counterpart, and this is coherent with El-Nakhel et al.’s [46] findings. The high accumulation of PO_4_, K and Cl in plants cultivated in 100% compost, and Mg and Ca in plants cultivated in 100% simulant, is mostly explained by the abundance of bioavailable fractions of these ions in the mixtures. As described in a complementary study by Caporale et al. [37], the concentrations of water-soluble K, Cl, NO_3_, PO_4_ and SO_4_ in the 100% MMS-1 substrate were less than 4% of the concentrations of the same nutrients in the 100% compost substrate, while Mg and Ca were 13% and 17% less, respectively, indicating the good bioavailability of the two nutrients—even in the pure MMS-1 substrate. Clearly, compost affects plant mineral content [47,48,49]. Indeed, Ca, Mg and Na contents showed a lower accumulation in the presence of compost in the mixtures, which might be due to the cation exchange capacity of the compost regulating the release of the elements from the substrates to the plants. On the other hand, although MMS-1 was found to be very rich in Al oxides [37], Salanova plants did not show any Al phytotoxic effect, since this element is poorly soluble and poorly bioavailable in sub-alkaline growth substrates such as those used in the experiment, whilst it exerts phytotoxicity at highly acidic pHs, with soluble cations undergoing acid hydrolysis [50]. Despite this, in the 100% simulant, green and red Salanova plants grew respectively less by 20.6% and 22.6% in comparison to the 30:70 mixture. This can be justified by the lower NO_3_, PO_4_ and K shoot concentrations and PO_4_ and K root concentrations compared to other mixtures. Besides a higher content of nutrient, MMS-1 amended with compost also had enhanced physical (bulk density and pore-size distribution) and hydraulic (water holding capacity and retention) properties compared to the pure simulant, which may have positively influenced the crop performance [37]. In particular, it was evident that the compost addition to the simulant proportionally increased the amount of water retained by the substrate and enhanced more macropore and micropore domains [37]. The decrease in K shoot and root concentrations were inversely correlated with Na shoot and root concentrations. This behavior can be interpreted as a result of a K shortage, with Na substituting K in non-specific functions like vacuolar osmotic potential maintenance [27]. Accordingly, Caporale et al. [37] supposed that the consistent bioavailable pool of Na in MMS-1, together with an alkaline pH and the absence of biological fertility, could have induced a salt stress in plants grown in pure simulant substrate. Furthermore, Salanova nitrate content expressed on a fresh weight basis in all four mixtures was within the maximum nitrate limit for lettuce set by the European Commission Regulation No 1258/2011 for commercialization. 

Red Salanova showed a higher content of lutein, β-carotene and total chlorophyll in comparison with the green cultivar, which is in harmony with El-Nakhel et al.’s [46,51] results. Nevertheless, only lutein was ameliorated by the presence of the compost, in mixtures 30:70 and 70:30, respectively. These findings are not fully in line with Thatikunta et al. [52] and Ouni et al. [41] who declared that organic matter can increase chlorophyll and carotenoid content. Contrastingly, Lesfrud et al. [53], Kolton et al. [54] and Ouzounis et al. [55] declared that chlorophyll, lutein and β-carotene are mainly influenced by light. 

Moreover, total ascorbic acid, other than being more concentrated in red Salanova, was the highest in the 30:70 mixture and 100% simulant for this cultivar, probably because the lower chemical and biological fertility of the two simulant-rich substrates caused a greater oxidative stress in the plants. As for total polyphenols, these were also highly rich in red Salanova (around 123% more than in the green cultivar) and positively modulated with the increase in the compost percentage in the mixture, while they remained statistically equal in green Salanova among all four mixtures. Such a diverse modulation pattern of polyphenols in both cultivars was also noted in El-Nakhel et al.’s [27] work, where green and red Salanova were subjected to a nutrient solution eustress. The antioxidant activity of plants is affected by the amount of organic matter present in the substrate, namely the compost rate in our experiment, due to various factors such as higher K availability since this element is strongly linked to enzymatic activities [56,57], the greater abundance of soluble salts [58] and micronutrients [59]. As matter of fact, our results showed a positive correlation between the compost rate in the substrate (S) and total polyphenols (r > 0.95), confirming the potential qualitative improvement of vegetables due to compost application, as reported by Sousa et al. [60], Saikia and Upadhyaya, [61], Aminifard et al. [62] and Lujàn-Hidalgo et al. [63]. The relevant presence of aromatic moieties and, hence, of stable and humified organic compounds in the compost, evidenced by Caporale et al. [37] through infrared spectroscopy and thermogravimetric analysis, may have stimulated the production of polyphenolic compounds in lettuce foliar biomass [64,65].

Overall, red Salanova had a better phytonutrient profile in comparison to its green counterpart, notwithstanding the mixture adopted. Such dense bioactive profile was as well proven for red Salanova in previous studies [9,21,27,38,51]. Similarly, the study of Neocleous et al. [66] showed that red “baby” lettuce exhibited better antioxidant activity in comparison to green “baby” lettuce when subjected to saline stress. Indeed, as declared by Rapisarda et al. [67] and Rouphael et al. [68], it is the genotype and the extrinsic stressors that affect the formation of bioactive compounds.

## 4. Materials and Methods 

### 4.1. Plant Growth Conditions and Experimental Design

A nineteen-day experiment was carried out in a phytotron open gas exchange climate chamber (28 m^2^: 7.0 × 2.1 m × 4.0 m; W × H × D) at the experimental farm of the Department of Agricultural Sciences, University of Naples Federico II, Italy. The adopted temperature regime was 24/18 °C light/dark, respectively, while relative humidity ranged between 65% and 75% and was maintained through a fog system. High-pressure sodium (HPS; Master SON-T PIA Plus 400 W, Philips, Eindhoven, The Netherlands) lamps were used to provide a 12 h photoperiod and 420 µmol m^−2^ s^−1^ light intensity at canopy level. Ambient CO_2_ concentration (370-410 ppm) was adopted for this experiment, while air dehumidification and circulation were maintained by two heating, ventilating and air conditioning (HVAC) systems. 

Green and red Salanova^®^ (Rijk Zwaan, Der Lier, The Netherlands) were the chosen butterhead lettuce cultivars (*Lactuca sativa* L. var. *capitata*). Fourteen days after sowing, these cultivars were transplanted in pots (7 × 8 × 8 cm) filled with one of four different substrate mixtures, as follows: 100:0, 70:30 30:70 and 0:100 v:v of MMS-1 and compost. The Mojave Mars simulant (MMS-1) was bought from The Martian Garden (Austin, Texas, USA), while the compost made from vegetal waste was bought from GARDEA (Villafranca di Verona, Verona, Italy). The latter was sifted through a 2-mm sieve before the preparation of the mixtures. The mineralogical and physico-chemical properties of both mineral and organic substrates of the four mixtures are reported in Caporale et al.’s [37] study. 

The pots were distributed on propylene gullies, with a resulting density of 15.5 plants m^−2^ (43 cm inter-raw and 15 cm intra-row spacing). The plants were fertigated through a drip irrigation system (open loop) equipped with 2 L h^−1^ auto-compensating drippers. The nutrient solution consisted of a modified Hoagland formulation: 9.0 mM nitrate, 2.0 mM sulfur, 1.0 mM phosphorus, 4.0 mM potassium, 4.0 mM calcium, 1.0 mM magnesium, 1.0 mM ammonium, 15.0 µM Iron, 9.0 µM manganese, 0.3 µM cupper, 1.6 µM zinc, 20.0 µM boron, and 0.3 µM molybdenum. The pH and the electrical conductivity (EC) were 5.8 and 1.5 dS m^−1^, respectively.

A factorial combination of four different substrate mixtures and two lettuce cultivars with different pigmentations accounted for eight treatments replicated three times. A randomized complete-block design was adopted for this experiment, with a total of 24 experimental units of seven plants each (a total of 168 plants).

### 4.2. Leaf Gas Exchange

A portable gas exchange analyzer (LCA-4; ADC BioScientific Ltd., UK) was used to measure the net CO_2_ assimilation rate (A_CO2_), stomatal resistance (r_s_) and transpiration rate (E) just before harvesting. Based on Carillo et al.’s [69] method, A_CO2_ was divided by E in order to calculate the Intrinsic Water Use Efficiency (WUEi). Fully expanded leaves were chosen to carry the measurements of the leaf gas exchange, and eighteen measurements were taken.

### 4.3. Fresh Biomass and Sampling

At harvest, shoot fresh weight (g plant^−1^) was determined on five plants per experimental unit. Then leaves were dried for 72 h in a forced-air oven set at 70 °C in order to determine the dry matter percentage needed for the calculation of leaf nitrate content expressed per fresh weight. Corresponding roots were washed with distilled water and placed in the oven to obtain the dry material necessary for mineral analysis. Two plants per experimental unit were directly frozen in liquid nitrogen, lyophilized and stored at -80 °C for phytochemical analysis. 

### 4.4. Total Nitrogen, Nitrate and Mineral Content 

Dried leaves and roots were ground in a Wiley mill. For foliar total nitrogen determination, the Kjeldahl method was employed [70], using 1 g of dried samples. As for foliar and root mineral content determination, 0.25 g of the dried material was analyzed by ion chromatography (ICS-3000, Dionex, Sunnyvale, CA, USA) based on the method adopted by Rouphael et al. [71]. 

### 4.5. Total Chlorophyll and Total Ascorbic Acid Content

Total chlorophyll and total ascorbic acid content (TAA) were assessed by UV–Vis spectrophotometric analysis based on Lichtenhaler and Wellburn [72] and Kampfenkel et al.’s [73] protocols, respectively. Fresh lettuce material was used for both protocols. After extraction, a spectrophotometer (Hach DR 2000, Hach Co. Loveland, CO, USA) was used to measure the absorbance at 647, 664 and 525 nm, in order to determine chlorophylls a, b and TAA, respectively. Total chlorophyll was calculated as the sum of chlorophylls a and b. 

### 4.6. Carotenoids Quantification by HPLC-DAD and Polyphenols Analysis by UHPLC-Q-Orbitrap HRMS

As described in Kyriacou et al. [74], carotenoids were extracted from freeze-dried lettuce material in ethanol enclosing 0.1% butylated hydroxytoluene (BHT) using the altered method of Kim et al. [75] and quantified by HPLC-DAD (diode-array detector). 

As for polyphenols, a UHPLC system (UHPLC, Thermo Fisher Scientific, Waltham, MA, USA) was used for quantification and separation. A Q Exactive Orbitrap LC–MS/MS (Thermo Fisher Scientific, Waltham, MA, USA) was used to facilitate the analysis of the mass spectrometry. The details of the polyphenol extraction are described by Kyriacou et al. [76].

### 4.7. Statistical Analysis

The obtained data were subjected to an analysis of variance (two-way ANOVA) using the software package SPSS 20. The mean effect of simulant:compost and the interaction between the two factors were obtained using Duncan’s Multiple Range Test (DMRT), performed at *P* ≤ 0.05. Furthermore, Student’s *t*-test was used to compare the two cultivars of lettuce.

## 5. Conclusions

Future space missions intended for the colonization of Mars are partnered with economical and mechanical constraints when considering a replenishment from Earth. Such a fact could be drastically alleviated by enhancing in situ resource utilization, like the opportune exploitation of Mars regolith as the main substrate for vegetable production. The physical, chemical and hydraulic attributes of this substrate, also known as Mars soil, can be improved by the addition of organic residues produced in situ, which can evoke a better quality and higher yield from the produced vegetables. Red Salanova presented a higher yield, photosynthetic activity and bioactive compounds in comparison to its green counterpart. The 30:70 (simulant:compost) mixture was demonstrated to be the most convenient mixture in terms of increasing yield, A_CO2_, WUEi, total ascorbic acid and total polyphenols of the red cultivar. The cultivation on 100% simulant substratum was feasible as well, although it yielded around 20% less in terms of production and displayed a decrease in NO_3_, PO_4_, K and bioactive compounds in the shoots, except for total ascorbic acid. In spite of these evidences, the 70:30 mixture represents a more realistic scenario when taking into consideration the sustainable use of compost as a limited resource in space farming, while accepting a slight significant decline in yield and quality in comparison to the 30:70 mixture. These findings reassure space explorers concerning the utility of Mars regolith as a cultivation substrate and demonstrate the importance of using the organic residues produced by any cultivation in space in order to enhance the fertility of this mineral substrate. Nevertheless, future studies regarding cultivations without additive fertigation and solely counting on in situ fertility are of major importance in order to reduce any additional load; moreover, testing organic matter from conveniently treated human excrement is also worthy of investigation.

## Figures and Tables

**Table 1:** Shoot and root mineral composition of green and red Salanova lettuce as influenced by substrate mixtures (four different rates of MMS-1 simulant:compost v:v).

Source of Variance	NO_3_ (g kg^−1^ dw)		PO_4_ (g kg^−1^ dw)		K (g kg^−1^ dw)		Ca (g kg^−1^ dw)		Mg (g kg^−1^ dw)		Na (g kg^−1^ dw)		Cl (g kg^−1^ dw)		SO_4_ (g kg^−1^ dw)	
	Shoot	Root	Shoot	Root	Shoot	Root	Shoot	Root	Shoot	Root	Shoot	Root	Shoot	Root	Shoot	Root
Cultivar (C)																
Green Salanova	27.6	42.9 a	9.0	5.7	64.7	50.6	7.1	6.2	2.5	2.8	1.0	5.9	3.3	2.2	1.5 b	8.9
Red Salanova	30.4	28.8 b	10.4	7.5	71.7	44.6	6.2	6.0	2.5	2.9	1.0	5.2	3.1	1.9	2.5 a	9.4
Simulant:compost (v:v) (S)																
0:100	29.4 a	33.3 ab	11.2 a	8.7 a	82.7 a	69.2 a	4.8 c	5.6 ab	2.2 b	2.2 c	0.8 b	2.0 c	6.9 a	2.8 a	2.2 a	9.6 ab
30:70	32.5 a	24.9 b	11.9 a	7.3 a	75.2 b	48.4 b	6.4 b	6.7 a	2.4 b	2.5 bc	0.8 b	2.7 bc	2.0 b	1.7 b	2.3 a	9.3 b
70:30	32.4 a	43.6 a	9.5 b	7.3 a	69.5 c	57.4 b	6.9 b	6.6 a	2.3 b	3.0 b	0.9 b	4.8 b	2.0 b	2.0 b	2.0 a	11.1 a
100:0	21.7 b	41.6 a	6.2 c	2.9 b	45.2 d	15.5 c	8.5 a	5.3 b	3.2 a	3.9 a	1.7 a	12.8 a	1.7 b	1.9 b	1.4 b	6.6 c
C × S																
Green Salanova × 0:100	28.0	37.3	10.6	6.9	80.2	77.6	5.5	5.6	2.3	2.2 c	0.9	1.9	7.5	3.1	1.7	10.1
Green Salanova × 30:70	32.6	30.7	11.2	6.1	71.3	48.0	6.4	6.6	2.3	2.2 c	0.8	2.6	1.9	1.5	1.7	8.8
Green Salanova × 70:30	30.4	52.1	8.3	6.6	64.6	59.0	7.3	6.6	2.3	2.6 bc	1.0	5.0	2.2	2.2	1.6	10.5
Green Salanova × 100:0	19.5	51.5	5.8	3.2	42.6	17.8	9.3	5.9	3.2	4.3 a	1.5	14.0	1.7	2.2	1.1	6.2
Red Salanova × 0:100	30.9	29.2	11.8	10.6	85.3	60.8	4.2	5.6	2.1	2.3 c	0.8	2.1	6.4	2.4	2.8	9.1
Red Salanova × 30:70	32.4	19.1	12.8	8.6	79.2	48.9	6.3	6.9	2.6	2.8 bc	0.7	2.8	2.2	1.8	2.9	9.7
Red Salanova × 70:30	34.4	35.0	10.8	8.0	74.5	55.9	6.5	6.7	2.3	3.3 b	0.7	4.5	1.9	1.8	2.4	11.8
Red Salanova × 100:0	23.8	31.7	6.5	2.7	47.8	13.1	7.7	4.7	3.3	3.4 b	1.9	11.6	1.8	1.7	1.7	7.1
Significance																
Cultivar (C)	ns	**	ns	ns	ns	ns	ns	ns	ns	ns	ns	ns	ns	ns	***	ns
Substrate (S)	*	*	***	***	***	***	***	*	***	***	**	***	***	**	***	***
C × S	ns	ns	ns	ns	ns	ns	ns	ns	ns	*	ns	ns	ns	ns	ns	ns

Non-significant (ns). *, **, *** Significant at *P* ≤ 0.05, 0.01, and 0.001, respectively. Cultivar means were compared by *t*-test. Substrate mixture means and interaction were compared by Duncan’s multiple-range test (*P* = 0.05). Different lowercase letters within each column indicate significant differences (*P* ≤ 0.05).

**Table 2:** Total nitrogen, nitrate, total chlorophyll, lutein and β-carotene of green and red Salanova lettuce as influenced by substrate mixture (four different rates of MMS-1 simulant:compost v:v).

Source of Variance	Total N	Nitrate	Total Chlorophyll	Lutein	β-Carotene
	(g 100g^−1^ dw)	(mg kg^−1^ fw)	(mg 100g^−1^ fw)	(mg kg^−1^ dw)	(mg kg^−1^ dw)
Cultivar (C)					
Green Salanova	3.9	1488	10.3 b	85.5 b	262.4 b
Red Salanova	4.0	1528	21.8 a	249.5 a	511.2 a
Simulant:compost (v:v) (S)					
0:100	3.9	1542	15.4	170.3 b	386.9
30:70	4.0	1609	14.6	164.0 b	379.3
70:30	3.9	1637	16.7	199.4 a	437.3
100:0	3.8	1244	17.6	136.2 c	343.6
C × S					
Green Salanova × 0:100	3.9	1486	10.6	88.7	271.0
Green Salanova × 30:70	4.0	1670	10.2	88.8	262.7
Green Salanova × 70:30	3.9	1591	9.9	112.4	295.2
Green Salanova × 100:0	3.7	1205	10.5	52.1	220.7
Red Salanova × 0:100	4.0	1598	20.1	251.9	502.8
Red Salanova × 30:70	4.0	1548	19.0	239.3	495.8
Red Salanova × 70:30	4.0	1682	23.5	286.3	579.3
Red Salanova × 100:0	3.9	1283	24.7	220.3	466.6
Significance					
Cultivar (C)	ns	ns	***	***	***
Substrate (S)	ns	ns	ns	**	ns
C × S	ns	ns	ns	ns	ns

ns, **, *** Non-significant or significant at *P* ≤ 0.01, and 0.001, respectively. Cultivar means were compared by *t*-test. Substrate mixture means and interaction were compared by Duncan’s multiple-range test (*P* = 0.05). Different lowercase letters within each column indicate significant differences (*P* ≤ 0.05).

**Table 3:** Polyphenol profile of green and red Salanova lettuce, as influenced by substrate mixture (four different rates of MMS-1 simulant:compost v:v).

Source of Variance	Chlorogenic Acid	Caffeic Acid Hexoside	Caffeic Acid	Luteolin-7-Oglucoside	Apigenin Malonil Glucoside	Coumaroyl Quinic Acid	Coumaric Acid	Feruloyl Quinic Acid	Quercetin-3-O-Galactoside	Dicaffeoylquinic Acid	Quercetin-3-O-Glucuronide	Quercetin-3-O-Glucoside	Feruloylglycoside	Kaempferol-7-O-Glucoside	Rutin	Quercetin Malonylglucoside	Kaempferolo-3-O-Rutinoside	Feruloyltartaric Acid	Caffeoylferuloylquinic Acid
	(µg g^−1^ dw)																		
Cultivar (C)																			
Green Salanova	330 b	9.7	15.1 b	4.1 b	64.8 a	420.6 b	9.5 a	17.8 b	7.7 b	nd	69.3 a	7.4 b	10.0 a	4.1 b	814.0 b	614 b	51.8 b	1064 a	571 b
Red Salanova	4156 a	6.9	57.9 a	8.4 a	24.0 b	746.7 a	6.8 b	25.8 a	40.1 a	90.0	52.8 b	34.6 a	7.3 b	9.2 a	866.3 a	1276 a	73.3 a	978 b	656 a
Simulant:compost (v/v) (S)																			
0:100	2437 a	12.6 a	34.2 b	5.7 c	100.8 a	534.1 c	8.0 b	21.5 b	23.2 b	134.9	76.3 a	25.3 a	8.2 c	5.4 c	943.0 a	1335 a	67.4 a	1039 a	634 a
30:70	2534 a	6.8 c	46.4 a	6.6 b	26.0 b	620.8 b	7.7 b	23.7 a	34.4 a	73.5	73.6 a	21.3 c	9.5 b	6.8 b	846.2 b	914 b	60.3 b	1015 b	618 b
70:30	2345 a	7.4 b	48.3 a	7.5 a	32.4 b	502.9 c	9.2 a	25.1 a	22.7 b	84.0	60.2 b	22.8 b	10.8 a	9.1 a	808.1 c	774 b	62.0 b	1014 b	606 c
100:0	1658 b	6.4 c	17.1 c	5.1 d	18.5 c	676.6 a	7.8 b	16.8 c	15.2 c	67.5	34.1 c	14.6 d	6.1 d	5.4 c	763.3 d	757 b	60.5 b	1016 b	596 d
C × S																			
Green Salanova × 0:100	138 d	17.8 a	6.1 f	4.5 e	175.5 a	372.3 e	9.5 b	15.3 c	6.2 f	nd	101.3 a	5.8 f	8.7 c	4.2 d	883.8 b	865	61.2 b	1099 a	577 e
Green Salanova × 30:70	241 d	6.7 b	15.6 de	3.9 fg	30.4 bc	351.5 e	8.9 c	19.6 b	7.0 f	nd	74.9 b	6.5 ef	11.0 b	3.9 d	782.1 d	631	47.2 c	1051 b	573 ef
Green Salanova × 70:30	639 d	7.5 b	26.0 c	4.3 ef	31.9 b	396.0 e	10.7 a	21.3 b	10.3 e	nd	68.3 b	10.2 d	12.7 a	4.4 d	825.9 c	535	48.8 c	1054 b	569 f
Green Salanova × 100:0	302 d	7 b	12.7 ef	3.6 g	21.6 cd	562.4 d	8.9 c	15.1 c	7.2 f	nd	32.5 d	7.1 e	7.5 d	3.9 d	764.4 d	424	49.8 c	1054 b	566 f
Red Salanova × 0:100	4735 a	7.4 b	62.4 b	7.0 c	26.1 bc	696.0 c	6.5 e	27.8 a	40.3 b	134.9 a	51.2 c	44.8 a	7.7 d	6.7 c	1002.1 a	1805	73.6 a	980 c	692 a
Red Salanova × 30:70	4826 a	7 b	77.1 a	9.2 b	21.7 cd	890.2 a	6.6 e	27.9 a	61.9 a	73.5 c	72.2 b	36.1 b	8.0 d	9.6 b	910.3 b	1196	73.4 a	979 c	664 b
Red Salanova × 70:30	4050 b	7.3 b	70.7 a	10.7 a	32.9 b	609.8 d	7.6 d	29.0 a	35.1 c	84.0 b	52.1 c	35.5 b	8.8 c	13.7 a	790.4 d	1013	75.1 a	975 c	644 c
Red Salanova × 100:0	3014 c	5.8 c	21.5 cd	6.5 d	15.4 e	790.8 b	6.6 e	18.6 b	23.2 d	67.5 c	35.7 d	22.0 c	4.7 e	6.9 c	762.3 d	1090	71.1 a	978 c	625 d
Significance																			
Cultivar (C)	***	ns	***	***	*	***	***	***	***	na	ns	***	**	***	ns	***	***	***	***
Substrate (S)	**	***	***	***	***	***	***	***	***	***	***	***	***	***	***	***	***	***	***
C × S	***	***	***	***	***	***	*	**	***	***	***	***	***	***	***	ns	***	**	***

Non-significant (ns). *, **, *** Significant at *P* ≤ 0.05, 0.01, and 0.001, respectively; not detected (nd); not applicable (na). Cultivar means were compared by *t*-test. Substrate mixture means and interaction were compared by Duncan’s multiple-range test (*P* = 0.05). Different lowercase letters within each column indicate significant differences (*P* ≤ 0.05).
